# Evaluation of the psychometric properties of the Swiss French version of the Older People’s Quality of Life questionnaire (OPQOL-35-SF)

**DOI:** 10.1186/s12955-022-01950-w

**Published:** 2022-03-09

**Authors:** Sophie Carrard, Claudia Mooser, Roger Hilfiker, Anne-Gabrielle Mittaz Hager

**Affiliations:** 1 School of Health Sciences, Physiotherapy, HES-SO Valais-Wallis, Rathausstrasse 25, 3954 Leukerbad, Switzerland; 2 Institut Notre-Dame de Lourdes, 3960 Sierre, Switzerland; 3 School of Health Sciences, Physiotherapy, Caphri-Care and Public Health Research Institute and HES-SO Valais-Wallis, Rathaustrasse 25, 3954 Leukerbad, Switzerland

**Keywords:** Older people, Quality of life, Questionnaire, Psychometric properties

## Abstract

**Background:**

The proportion of the world population aged over 65 years is increasing in the world population. Quality of life is an important factor in the biopsychosocial management of older patients. The Older People’s Quality of Life-35 (OPQOL-35) questionnaire was developed specifically for assessment of the quality of life of older people. The aim of this study is to evaluate the psychometric properties of a Swiss French version of the OPQOL-35 questionnaire (OPQOL-35-SF).

**Methods:**

Forward–backward procedure was used to translate the original questionnaire from English into Swiss French. A sample of older people then completed the questionnaire. Construct validity of the OPQOL-35-SF was evaluated by comparing the results with those from three other questionnaires [World Health Organisation Quality of Life in older people questionnaire (WHOQOL-OLD), Control, Autonomy, Self-realization, Pleasure in 12 questions (CASP-12), and EuroQol-5-dimensions-5-levels (EQ-5D-5L)] and two visual analogue scales (health and quality of life). The structure of the OPQOL-35-SF questionnaire was assessed using exploratory and confirmatory factor analysis. To evaluate the reliability the OPQOL-35-SF questionnaire was completed a second time after 7–23 days.

**Results:**

A total of 264 older people completed all the questionnaires at the first session, and 262 completed the OPQOL-35-SF again at the second session. Mean age of participants was 76.8 (standard deviation (SD) = 7.1) years. The majority of participants were women (73.9%). The Kaiser–Meyer–Olkin Measure of Sampling Adequacy (KMO) was 0.86 and Bartlett’s test of sphericity was significant (*p* < 0.001). The result of Exploratory Factor Analysis (EFA) revealed 8 factors with eigenvalues greater than one, which explained 58% of the observed variance. All items had an acceptable loading (< 0.30) in at least one factor. The convergent validity presented low to moderate correlations (rho: 0.384–0.663). Internal consistency was good (Cronbach’s alpha 0.875 for test and 0.902 for retest). Test–retest reliability presented an intra-class correlation coefficient, two-way random effects, absolute agreement, single rater (ICC_2.1_) of 0.83 [95% confidence interval (CI) 0.78–0.87].

**Conclusions:**

The Swiss French version of the OPQOL-35 questionnaire shows good psychometric properties, which permit its use in clinical practice or research. A supplementary sample would be necessary for a better distribution of the items in the different factors.

**Supplementary Information:**

The online version contains supplementary material available at 10.1186/s12955-022-01950-w.

## Background

The world population is ageing at an increasing rate. This acceleration in population ageing will impact almost all aspects of society, and, in August 2020, the World Health Assembly endorsed the “United Nation (UN) Decade of healthy ageing 2021–2030” [1]. It is estimated that, between 2015 and 2050, the proportion of the world’s population over 60 years of age will almost double, from 12 to 22% [2]. In the European Union, the proportion of people over 60 years of age was approximately 15% in 2014 and could reach 30% by 2050 [3].

Ageing is associated with declining health [4] and is often related to multiple chronic and acute diseases [5]. This places a high burden on the health care system, both in hospitals and in community care [6]. Due to the ever-increasing costs of health care, the mean length of stay in hospital for older patients is decreasing [7]. Home-based care is therefore increasingly required, to provide assistance with daily tasks and enable older adults to live at home [8, 9]. In 2018, 1.5% of Swiss people aged between 65 and 79 years, and 15.3% of those aged over 80 years, were living in a health care institution [10]. In future, older people, whether healthy or not, are increasingly likely to live at home [11]. In addition to their caring role, a goal for caregivers is to enhance quality of life (QoL) [12]. Maintaining QoL is one of the most important outcomes of care services for older adults [8]. The measurement of QoL may help to predict adverse health outcomes, such as death and nursing home placement, in older people, even after adjustment for frailty [13]. However, it is not clear how QoL should be defined and assessed in older people living at home.

QoL can inherently be defined as “*a dynamic, multi-level and complex concept, reflecting objective, subjective, macro-societal, and micro-individual, positive and negative influences which interact*” [14]. QoL is also a network of objective and subjective factors, that includes relationships between psychological and social indicators, objective living conditions and subjective well-being [15]. In a recent thematic synthesis, Van Leeuwen et al. described and categorised the aspects of QoL into nine domains and 38 subthemes [8].

There is a multitude of questionnaires for evaluation of QoL, some of which have been developed specifically for older adults [16]. The most used questionnaires in this field differ in the number of dimensions analyzed as well as in the number of items. The World Health Organisation Quality of Life in older people questionnaire (WHOQOL-OLD) comprises 24 items distributed in six dimensions [17]. The Control, Autonomy, Self-realization, Pleasure (CASP) questionnaires evaluate four dimensions, as stated in the name, and comprise 19 (CASP-19) [18] or 12 (CASP-12) items [19]. The World Health Organisation Quality of Life in the ageing population questionnaire (WHOQOL-AGE), which has two dimensions and 30 items [20], was constructed with five items from the WHOQOL-OLD and eight items from the European Health Interview Survey-Quality of Life (EUROHIS-QOL) [21]. The Older People’s Quality of Life-35 (OPQOL-35) questionnaire [22] comprises 35 items in eight dimensions, and has a brief version (OPQOL-brief) with 13 items [23].

Most of these questionnaires were first conceptualized and validated in English, and some of them have been translated into other languages. To our knowledge, the OPQOL-35 has been translated and validated for use in Iran [24], Czech Republic [25], China [26] and Uganda [27]. It has also been used in studies in Albania [28], India [29], Sri Lanka [30], Pakistan [31], Malayesia [32] and Indonesia [33]. Some countries, such as Turkey [34], Iran [35] and Norway [36], have translated and used the OPQOL-brief with 13 items. Although French is spoken by approximately 300 million people worldwide, making it the fifth most widely used language [37], the OPQOL-35 is not yet available in French. The aim of this study is to evaluate the psychometric properties of the Swiss French version of the Older People’s Quality of Life questionnaire (OPQOL-35-SF).

## Methods

### Original version of the OPQOL questionnaire

In 2009, Ann Bowling developed the OPQOL-35 [22, 38, 39]. It consists of 35 statements for which older people indicate their agreement by selecting from the following options: “*strongly agree*”, “*agree*”, “*neither agree nor disagree*”, “*disagree*”, “*strongly disagree*” or by giving a score of 1–5. A higher score represents better QoL, and scoring requires the reverse coding of positive items. The total score ranges from 35 (worst possible QoL) to 175 (best possible QoL). The questionnaire covers eight domains: (a) Life overall (4 items), (b) Health (4 items), (c) Social relationships and participation (8 items), (d) Independence, control over life and freedom (5 items), (e) Home and neighborhood (4 items), (f) Psychological and emotional well-being (4 items), (g) Financial circumstances (4 items) and (h) Culture and religion (2 items). The psychometric proprieties of the original English version of the OPQOL-35 were analysed by Bowling [22]. Cronbach’s alpha ranged between 0.70 and 0.90 for internal consistency without item redundancy. Test–retest correlations (over a period of four weeks) ranged from moderate to high (r 0.403–0.782). Convergent construct validity was tested with CASP-19 [18] and WHOQOL-OLD [17]. OPQOL-35 showed moderate to high correlations with these two questionnaires (rho 0.380–0.732, *p* < 0.01) for total scores.

There was no consensus regarding the optimal factorial structure of the questionnaire. Although the English version includes eight dimensions, principal components analysis (PCA) mainly identified two or four dimensions [22], although another analysis identified nine dimensions [39]. The authors of the Chinese and Persian translated versions of OPQOL-35 identified eight dimensions [24, 26], while the authors of the Czech translation estimated seven dimensions as optimal [25].

### Swiss French version of the OPQOL questionnaire

With the consent of the author of the original version, Ann Bowling, a research team translated the OPQOL-35 questionnaire into Swiss French according to current guidelines [40]. A health professional and a naive translator separately translated forward the English version into Swiss French [respectively translation 1 (T1) and translation 2 (T2)], both were native French speakers Together, the two translators and a recording observer produced a synthesis of the translation, resulting in a first Swiss French version of the questionnaire (T-12). Two native English speakers back-translated T-12 into English. Neither were informed of the concept being explored. Both back-translations (BT1 and BT2), both forward-translations (T1 and T2), T-12, and the original English version of the questionnaire were submitted to an expert committee to consolidate all the versions and develop a pre-final version of the Swiss French OPQOL-35. The expert committee comprised the four translators, two health professionals, and a linguist. The pre-final version was then submitted to 19 older adults who gave comments. The feedback was included in the second pre-final version. Bütikofer and Rausis [41] applied the second prefinal version to 37 older people. Since no comprehension issues were pointed out, this version is considered the final Swiss French version (OPQOL-35-SF) (Additional file 1).

### Participants

AB, SC, CM and LR recruited older adults, aged 65 years or more, who were living in their own home and able to understand and write French language, from two French-speaking cantons of Switzerland (Vaud and Valais) during two periods: April–May 2017, and June-December 2018. No specific exclusion criteria were set. Cognitive impairment was not specifically ascertained, more than the ability to understand and write French, as the participants lived independently in their own homes. They were recruited from medical-social centres, physiotherapy practices, associations of elderly people, and through personal contacts.

Recommendations for sample size for exploratory factor analysis (EFA) differ widely in the literature: for example, from 50 to 1000 persons [42]; between five and ten individuals per item [43, 44]; or more than 100 [45]. A total of 200 people seems to be necessary for confirmatory factor analysis (CFA) [43]. Considering a minimum of 50 individuals [46] and between three and ten individuals per item [24], a total of seven to eight individuals were chosen per item, i.e. between 245 and 280 people.

### Measures

To evaluate the construct validity of the OPQOL-35-SF, total scores were correlated with the French versions of the Visual Analogue Scale (VAS), WHOQOL-OLD [47], CASP-12 [19] and EQ-5D-5L [48]. Authorisations have been received from the World Health Organisation (WHO) for the use of WHOQOL-OLD and from EuroQol for the EQ-5D-5L. CASP-12 is available for use free of permissions.

VASs are single-item self-reported measurement tools, which are often used in health care practice to assess pain [49], patient satisfaction [50], anxiety [51] and health-related QoL [52]. The scientific literature did not attribute one or more authors to VASs, but they seem to have been developed and then used empirically by physicians and caregivers [53]. The global QoL VAS is recommended for measuring global QoL in clinical trials and has shown good validity and excellent reliability [54]. The score is recorded on a horizontal 100-mm line ranging from 0 “*worst imaginable quality of life*” to 100 “*perfect quality of life*”.

The WHOQOL-OLD was developed from the WHOQOL-100, which is a questionnaire from the WHOQOL Group within the WHO [17]. It measures QoL with 24 items in six subscales (sensory abilities; autonomy; past, present and future activities; social participation; death and dying; and intimacy), with four items per subscale. Items are scored with reverse coding of positive responses, so that a higher score means a higher QoL between 24 (lowest possible QoL) and 120 (highest possible QoL). Response scales are all 5-point but vary in their wording (“*Not at all*” to “*An extreme amount*” / “*Completely*” / “*Extremely*”; “*Very poor*” to “*Very good*”; “*Very dissatisfied*” to “*Very satisfied*”; “*Very unhappy*” to “*Very happy*”).

CASP questionnaires were developed based on the theories of Maslow and Giddens about the satisfaction of human needs [18]. QoL is evaluated in four domains: control, autonomy, self-realization, and pleasure. The original version contains 19 items, and two short versions with 12 items have been developed: one in 2005, specifically for the Survey of Health, Aging and retirement in Europe [55] and a second one in 2008 [56]. Items are scored on a 4-point Likert response scale “*Often*”, “*Sometimes*”, “*Not often*” and “*Never”*, with reverse coding of positive responses, so that higher scores mean higher QoL. The CASP-12 scale ranges from 0 (complete absence of QoL) to 36 (total satisfaction in all four domains).

EuroQol Group developed the EQ-5D in the 1990s to evaluate QoL related to health, with 3 levels of answers (3L), and, in 2009, they added two levels to get five levels (5L) to improve the instrument’s sensitivity and reduce the ceiling effects. The tool comprises two parts: one for the descriptive system and one for the visual analogue scale (EQ VAS). The descriptive system comprises five dimensions: mobility, self-care, usual activities, pain/discomfort, and anxiety/depression. Each dimension has five levels: “*no problems*”, “*slight problems*”, “*moderate problems*”, “*severe problems*” and “*extreme problems*”. The EQ VAS records the patient’s self-rated health on a vertical VAS, where the Endpoints are labelled “*The best health you can imagine*” and “*The worst health you can imagine*” [57]. Scoring is calculated with an algorithm specific to each country.

### Data collection

The participants completed the questionnaires under the supervision of a research assistant (SC or CM), either individually or in a group. They completed the questionnaires on electronic tablets, laptops or in paper format, at the participant’s home or in another convenient location. To analyse test–retest reliability, the OPQOL-35-SF was administered twice, with an interval of 6–23 days. It has been shown previously that there is no significant difference, clinical or statistical, with an interval of two days or two weeks [58]. In some exceptional situations, and for logistical reasons, the questionnaire for the retest was given at the end of the first session with a pre-stamped and pre-addressed envelope. Participants were instructed to complete and return the questionnaire after seven days.

At the first session, the research assistant explained the study in detail. The participants provided information or answers in the following order: their personal data and general information about health status, questionnaires WHOQOL-OLD, CASP-12, EQ-5D-5L and OPQOL-35-SF. The first session lasted between 30 min (individual session) and two hours (group session). At the second session, the participants completed only the OPQOL-35-SF and answered the following question: “*Since our first meeting, have you experienced any events that could have influenced your quality of life*?”. If the answer was “Yes”, they were asked: “*Does this event influence your quality of life positively or negatively*?” and they were asked to describe the event. The second session lasted between 10 and 30 min.

Data were collected online using REDCap (Research Electronic Data Capture) software [59] and saved to a secure server at the University of Applied Sciences, Fribourg, Switzerland. All data were exported in Microsoft Excel to be cleaned, before analysis with the software R, version 3.5.2 (within R-Studio), and Stata version 15.1.

### Data analysis

Floor and ceiling effects were considered to be present if more than 15% of participants scored a total of 35 (the lowest possible score) or 175 (the highest possible score) on the OPQOL-35-SF.

*Construct validity *The factor structure of the OPQOL-35-SF was evaluated by performing EFA with varimax rotation [60]. This enabled the variables to be grouped by factors, and those that were not related to the construct to be eliminated [45, 61]. In brief, EFA measures the coefficient of variance of items between two populations. A large variance indicates a difference in the meaning of the question, which may be due either to the translation or to cultural variation [62]. Factor analysis can be exploratory or confirmatory; both can be complementary [45]. However, as the Czech questionnaire did not contain the same number of factors as the original English version, CFA was not possible. Therefore, it was decided to perform EFA to obtain the correct number of factors for the French translation. CFA was not performed on our sample, as it was not large enough to be separated into two distinct samples, and analysis on the same sample is irrelevant. For factor analysis, the Kaiser–Meyer–Olkin Measure of Sampling Adequacy (KMO) should exceed the threshold of 0.8 [63, 64] and the correlation matrix must contain correlations = 0 (*p* < 0.05) with the Bartlett’s Test of Sphericity [42, 65]. EFA enables identification of the different factors that define the construct [61]. There is no expectation regarding the nature and number of factors, and this helps to streamline questionnaires by grouping inter-correlated questions [42, 43, 45]. EFA is measured using Principal Component Analysis (PCA) and Varimax rotation. It is expressed by eigenvalues > 1.0 and variance coefficients > 0.40 from the correlation matrix [24, 26, 43]. The weight of the variables represents the correlation between the original variable and the factor. The weight should be greater than 0.35 for a sample of 250–350 individuals. Our analysis was based on a threshold of 0.30, as in the study of Bowling et al. [39]. Scree plots enabled identification of the ideal number of factors, either the one before the inflection point of the curve, or the one at the level of the ideal eigenvalue, equal to 1 [65].

*Convergent validity* was evaluated using Spearman’s rank correlations between scores of VAS for QoL, the OPQOL-35-SF, WHOQOL-OLD, CASP-12 and EQ-5D-5L, including the VAS for health [66]. Because the scoring scales of these questionnaires are different, for analysis, they all were converted to the scale used for OPQOL-35 (Additional file 2) for the analysis.

Cronbach’s alpha tests the strength of the association between each scale item and the full scale. It was used to evaluate *internal consistency* [66]. The closer the Cronbach’s alpha is to 1, the more reliable is the scale. It should be between 0.7 and 0.9 [22, 46, 67].

Intra-class correlation coefficient, two-way random effects, absolute agreement, single rater (ICC_2.1_) has been used to evaluate the *test–retest reliability* [68]. Terwee et al. [46] and De Vet et al. [62] consider an ICC of 0.70 as acceptable to demonstrate good reliability. Koo & Li [69] have suggested that ICC values < 0.5 indicate poor reliability, 0.5–0.75 moderate reliability, 0.75–0.9 good reliability, and > 0.90 excellent reliability. Agreement was analysed as percentage, with weighted Cohen's kappa coefficient and prevalence-adjusted bias-adjusted kappa (PABAK). The use of PABAK minimizes the influence of a difference of 1 in the answer, as the answers to the items range from 1 to 5 [70, 71]. Landis and Koch consider a score > 0.80 as almost perfect, and Fleiss considers a score > 0.75 as excellent [62].

To avoid *missing data* CM and SC checked the questionnaires as the participants completed them, so that missing answers could be completed. Only two VAS QoL answers and one answer regarding the occurrence of a fall in the last 12 months were still missing. For each analysis, all available data were used (pairwise deletion of cases).

## Results

### Sample characteristics

The participants’ characteristics are shown in Table 1. A total of 264 older people completed the questionnaires at the first session, and 262 completed the OPQOL-35-SF at the second session. The mean age of the 264 participants was 76.8 ± 7.1 (range 65–96) years, and 87.1% were native French speakers. The 34 non-native French speaker participants had been speaking French for a mean of 55 years. Most of the participants were women (73.9%), rural residents (67%), practiced physical activity (87.1%) and took medication (73.5%).

**Table 1:** Characteristics of participants (n = 264)

Age in years, mean (SD/range)	76.8 (7.1/65.0–96.6)
Women, n (%)	195 (73.9)
Native French speaker, n (%)	230 (87.1)
Living in the country, n (%)	177 (67)
Living in a building, n (%)	135 (51.1)
Living in couple, n (%)	135 (51.1)
BMI in kg/m^2^, mean (SD/range)	25.7 (4.2/16.4–41.0)
Physically active, n (%)	230 (87.1)
With pain, n (%)	122 (46.2)
With health problems, n (%)	95 (36)
Taking medicine, n (%)	194 (73.5)
With sight’s disorders, n (%)	159 (60.2)
With hearing’s disorders, n (%)	76 (28.5)
With balance disorders, n (%)	59 (22.1)
Fear of falling, n (%)	79 (29.6)
Walking aid outside, n (%)	50 (18.7)
Walking aid inside, n (%)	17 (6.4)
Fall in the last 12 months, n = 263, (%)	55 (20.9)

SD, standard deviation; n, number of participants

Twenty-four participants reported events that had strongly influenced their QoL between the first and second sessions. Their scores were excluded for the PCA of the OPQOL-35-SF retest (n = 238).

Item distribution showed no floor or ceiling effects.

### Construct validity

**Fig. 1:**
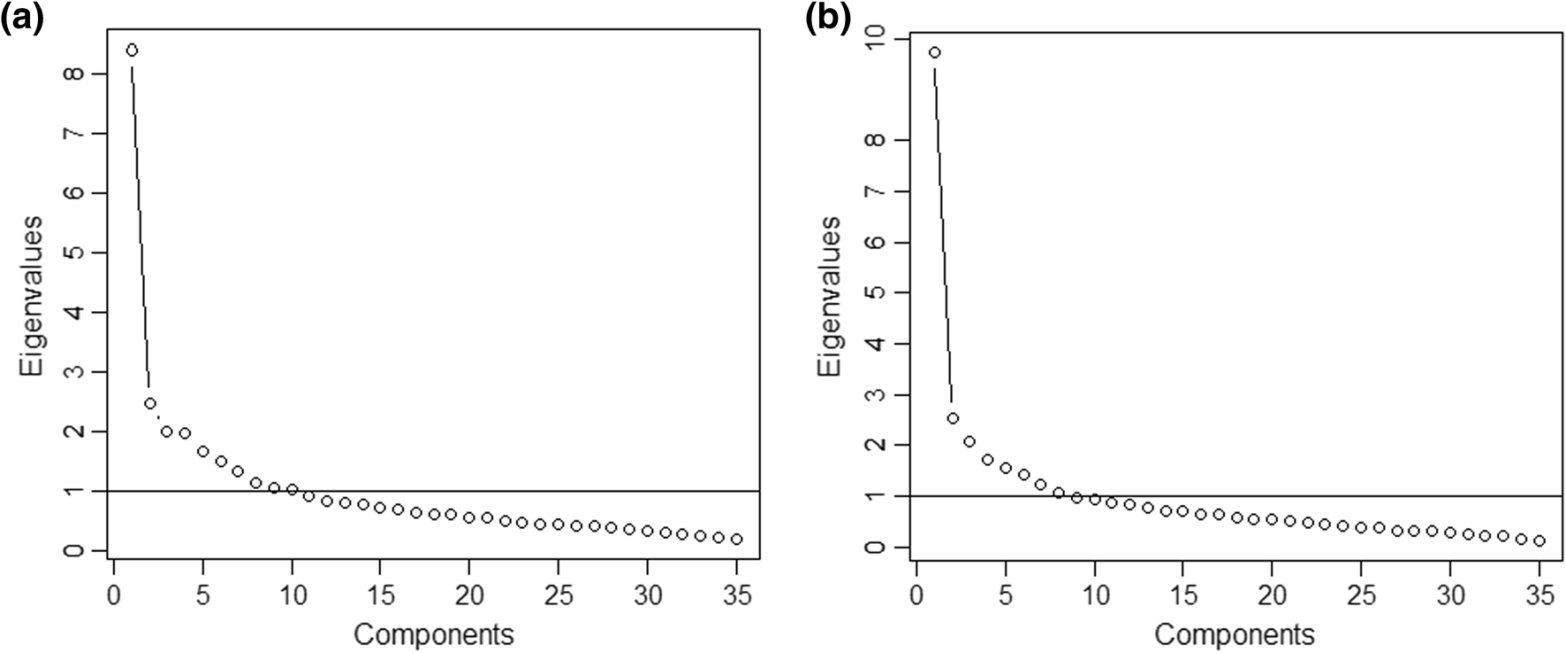
Scree plot of eigenvalues from the exploratory factor analysis. **a** OPQOL-35-SF test; **b** OPQOL-35-SF retest

EFA was performed to test the structural validity of the OPQOL-35-SF. The ratio of participants to items was 7.54:1. The KMO value of sampling adequacy was 0.86 for the OPQOL-35-SF test and 0.88 for OPQOL-35 retest, exceeding the recommended value of 0.8 [63, 64]. Bartlett’s Test of Sphericity was statistically significant for the OPQOL-35-SF test (Chi-square 3424.096, 595 degrees of freedom, *p* < 0.001) and for the OPQOL-35-SF retest (Chi-square 4117.709, 595 degrees of freedom, *p* < 0.001), supporting the factorability of the correlation matrix [63]. Eight factors were extracted and identified, using a minimal eigenvalue of 1 as the factor criterion. The eight factors explained 58% of the variance observed. Scree plots of the OPQOL-35-SF test and retest showed an ideal number of eight factors (Fig. 1a, b). This was more explicit in the test than in the retest.

**Fig. 2:**
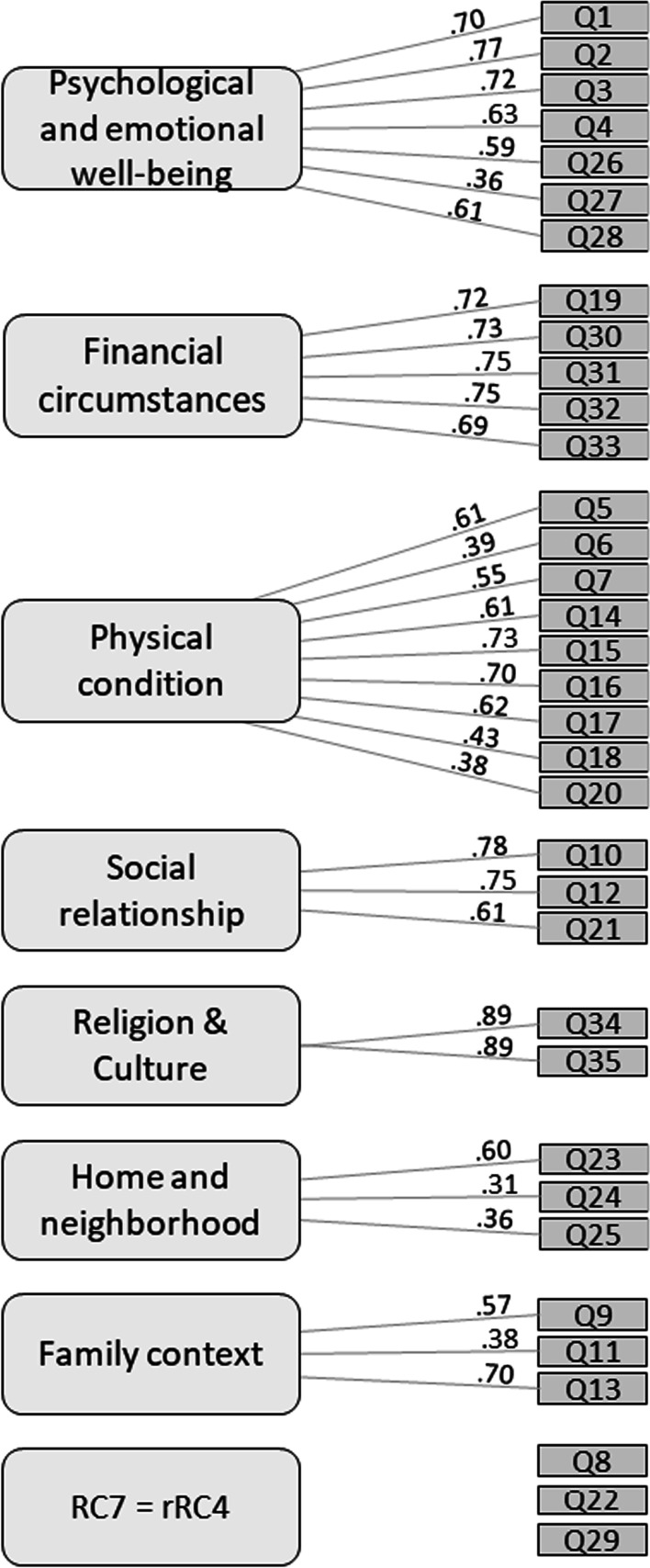
Factors’ structure of the Older People’s Quality of Life Questionnaire (OPQOL) derived from principal component analysis (PCA)

PCA and Varimax rotation for the OPQOL-35-SF test and retest (Additional file 3 and Additional file 4) presented the subdivision of the items with a significant weight (< 0.30) into eight factors. Component 1 explained the largest proportion of the variance for the test (0.21) and component 1 and 8 for the retest (0.19). In the Swiss French version of the questionnaire, the distribution of items in the dimensions (Fig. 2) differed from Bowling’s original English version [22]. The dimension “Life overall” disappeared, and its four items (Q1–Q4) were integrated into the dimension “Psychological and emotional well-being” with items Q26-Q28. Item Q19 *“The cost of the things compared to my pension/income restricts my life”* was integrated into the dimension "Financial circumstances" with items Q30–Q33. A new dimension, entitled “Physical condition”, was added, which included three items (Q5–Q7) from the original “Health” dimension, three items (Q14–Q16) from the original “Social relationships/leisure and social activities” dimension, and three items (Q17, Q18 and Q20) from the original “Independence, control over life, freedom” dimension. The original dimension “Social relations/leisure and social activities” was divided into two separate new dimensions: “Social relationship”, which included items Q10, Q12 and Q21, and “Family context”, which included items Q9, Q11 and Q13. Item Q22 *“I feel safe where I live”* disappeared from the dimension "Home and neighborhood". The dimension “Religion/culture” was unchanged. Finally, three items did not fit any of the identified dimensions: Q8 *“I am healthy enough to get out and about”*, Q22 *“I feel safe where I live”,* and Q29 *“If my health limits social/leisure activities, then I will compensate and find something else I can do”*. The final version is to find in Additional file 5.

Table 2 presents the scores of the different questionnaires measuring QoL, as original scores and transformed values (TV) for comparison with OPQOL-35. The average scores of the questionnaires, scaled to OPQOL-35, ranged from 142.2 ± 17.2 for CASP-12 to 155.4 ± 19.6 for EQ-5D-5L. The maximum score was reached in all questionnaires except for WHOQOL-OLD (which reached 118 out of 120).

**Table 2:** Scores of quality of life (QoL) questionnaires

	n	Mean	SD	Range	Mean TV (SD)
OPQOL-35-SF	264	147.91	13.43	109–175	–
VAS QoL	262	81.01	15.80	22–100	148.41 (22.109)
WHOQOL-OLD	264	97.81	10.11	71–118	142.64 (14.747)
CASP-12	264	27.58	4.42	15–36	142.24 (17.173)
EQ-5D-5L	264	0.786	0.214	− 0.033–1	155.43 (19.550)
VAS health (EQ-5D-5L)	264	77.88	16.98	26–100	144.04 (23.776)

n, number of participants; SD, standard deviation; TV, transformed values

### Convergent validity

Table 3 shows that OPQOL-35-SF (test), EQ-5D-5L, WHOQOL-OLD, CASP-12, VAS QoL and VAS health total score all correlated lowly to moderately with each other (r = 0.384–0.663; all *P* < 0.001) [72].

**Table 3:** Correlations between total scores of quality of life (QoL) questionnaires (Spearman’s rho)

	OPQOL-35-SF	VAS QoL	WHOQOL-OLD	CASP-12	EQ-5D-5L	VAS health
OPQOL-35-SF	–	0.561**	0.656**	0.663**	0.42**	0.425**
VAS QoL	0.561**	–	0.509**	0.54**	0.513**	0.661**
WHOQOL-OLD	0.656**	0.509**	–	0.655**	0.412**	0.384**
CASP-12	0.663**	0.54	0.655**	–	0.429**	0.467**
EQ-5D-5L	0.42**	0.513**	0.412**	0.429**	–	0.544**
VAS health	0.425**	0.661**	0.384**	0.467**	0.544**	–

OPQOL-35-S, Older People’s Quality of Live Questionnaire Swiss French; VAS QoL, Visual Analogue Scale for Quality of Life; WHOQOL-OLD, World Health Organisation Quality of Life in older people questionnaire; CASP-12, Control, Autonomy, Self-realization, Pleasure in 12 questions; EQ-5D-5L, EuroQol-5-dimensions-5- levels; VAS health, Visual Analogue Scale for health

**p < 0.001

### Internal consistency

Cronbach’s alpha for the total scale was 0.875 for the test and 0.902 for the retest. This shows good internal consistency [46] and may mean that the items evaluate the same construct [73].

### Test–retest reliability

A total of 262 older people completed the OPQOL-35-SF for the second time after an interval of 6–23 days. The mean scores of the total scale for the first and second tests were 147.91 (SD 13.43) and 146.03 (SD 14.28), respectively. ICC_2.1_ for the total sample (n = 262) was 0.83 (95% confidence interval (CI) 0.78–0.87), and ICC_2.1_ for the sample (n = 238) who did not report events that strongly influenced their QoL between the first and second sessions was 0.83 (95% CI 0.77–0.87). These results show a good reliability [46, 62]. The ICC_2.1_ of the subscales ranged between 0.58 and 0.84 for the older people without life changes, and between 0.59 and 0.82 for those who reported events that influenced their QoL (Table 4).

**Table 4:** Swiss French version of the OPQOL-35 questionnaire (OPQOL-35-SF) subscales test–retest reliability (ICC2.1)

Subscales	ICC_2.1_ (95% CI) (n = 262)	ICC_2.1_ (95% CI) (n = 238)
Life overall	0.65 (0.57–0.71)	0.63 (0.54–0.70)
Health	0.67 (0.58–0.74)	0.67 (0.58–0.74)
Social relationship/leisure and social activities	0.78 (0.73–0.83)	0.77 (0.72–0.82)
Independence, control over life, freedom	0.58 (0.50–0.66)	0.59 (0.50–0.67)
Home and neighborhood	0.68 (0.61–0.74)	0.68 (0.61–0.74)
Psychological and emotional well-being	0.68 (0.60–0.74)	0.66 (0.57–0.73)
Financial circumstances	0.73 (0.67–0.78)	0.72 (0.65–0.78)
Religion/culture	0.84 (0.80–0.87)	0.82 (0.77–0.86)

ICC_2.1,_ intraclass correlation coefficient; CI, confidence interval

Agreement between test and retest was 81.6–92.6% for the total sample and 81.6–93.3% for the reduced sample (sample without extra events between test and retest). Weighted Cohen’s kappa coefficients were 0.25–0.7 in the total sample and 0.22–0.67 in the reduced sample. Thirty items were rated as moderate in the total sample and 29 items in the reduced sample. PABAK was higher in the total sample than in the reduced sample: 0.63–0.85 and 0.63–0.87, respectively (Additional file 6).

## Discussion

The aim of this study was to evaluate the psychometric properties of the Swiss French version of the OPQOL-35 in older people in the French-speaking region of Switzerland. Political leaders as well as social and health professionals need effective and validated tools to assess QoL in older people [74, 75]. The results of this study demonstrate the good to very good psychometric quality of the OPQOL-35-SF questionnaire. It also showed the complexity of the subdivision of the QoL-items into pre-defined categories.

With 264 participants, the sample size in the current study was smaller than in studies evaluating the psychometric properties of the Czech [25], Persian [24], and Chinese [26] versions of the questionnaire. However, this sample size is sufficient to meet the requirements and recommendations for conducting a factor analysis [43].

EFA extracted and identified eight factors using a minimal eigenvalue of 1 as the factor criterion, which explained 58% of the variance observed. Like the original version by Bowling, the Persian version and the Chinese version, the Swiss French version of OPQOL-35 also has eight dimensions, while the Czech version has seven dimensions. Based on cross-cultural aspects reflected by the items, some dimensions of the original version have been renamed, some have fewer or more items, some dimensions have been integrated into others, and new dimensions have been created in the translated versions.

In the Iranian version, Bowling's "Life overall" dimension was retained, but it was expanded by the addition of two items *“I take life as it comes and make the best of things*” and “*I feel lucky compared to most people*” from the original "Psychological and emotional well-being" dimension. In the Swiss French version, the dimension "Life overall" was removed and its four items were integrated into the "Psychological and emotional well-being" dimension.

The Czech version added a new dimension entitled "Positive Approach", which includes the items “*I take life as it comes and try to make the best of it*”, “*I feel happy compared to most people*” and “*I tend to look on the bright side of the life*”.

The "Health" dimension of the original English version was removed from the Swiss French version. Three of its items were integrated into a new dimension, "Physical condition", which also included three items from the original dimension "Social relationship/leisure and social activities": “*I have social or leisure activities/hobbies that I enjoy doing*”, “*I try to stay involved with things*” and “*I do paid or unpaid work or activities that gives me a role in life*”. Similarly, three items from the original "Independence, control over life, freedom" dimension, namely “*I am healthy enough to have my own independence*”, “*I can please myself what I do*” and “*I have a lot of control over the important things in my life*” were incorporated into the "Physical condition" dimension. In the Chinese version, Chen et al. [26] created a new dimension entitled "Health and Independence". It seems that, for the Swiss French population 65 years old and over, the aspects of physical condition, or health, were closely related to independence, as they are for the Chinese population. This is similar to the Czech population, as, in their version, Mares et al. [25] created a dimension entitled "Health, independence, active life", which groups some items included in the "Physical condition" dimension of the Swiss French version.

The original English version of the OPQOL-35 includes a dimension entitled "Social Relationships/Leisure and Social Activities", which has been modified in all translated versions, both in its title and in the items attached to it. The Czech version has divided the items of this dimension into two new dimensions, a "Family and Safe Environment" dimension and a "Loneliness" dimension. In the Swiss French version, the items of Bowling’s original dimension were divided into a dimension entitled "Social Relations" and a new dimension entitled "Family Context" including the three items “*My family, friends or neighbors will help me if necessary*”, “*I have someone who gives me love and affection*” and “*I have my children around which is important*”. The notion of "Family" appears explicitly in the Czech version (Family and Safe Environment) and in the Swiss French version (Family context) while in the English, Iranian and Chinese versions, the items referring to it are distributed in different dimensions. In the Persian version, the item “*My family, friends or neighborhood will help me if necessary*” was not included in any of the questionnaire dimensions. The fact that the Chinese sample consisted exclusively of older people living alone could explain why the notion of "Family" was not highlighted in the Chinese version.

The three items *“I am healthy enough to get out and about”*, *“I feel safe where I live”* and *“If my health limits social/leisure activities, then I will compensate and find something else I can do”*, could not be attributed in any identified dimensions in the OPQOL-35-SF. Similarly, in the Persian version, Nikkhah et al. [24] were unable to include four items in the identified dimensions, namely “*My family, friends or neighbors would help me if needed*”, “*I can please myself what I do*”, “*The cost of things compared to my pension/income restricts my life*”, and “*I cannot afford to do things I would enjoy*”.

The OPQOL-35-SF correlated lowly with the EQ-5D-5L (r = 0.42, *P* < 0.001) and its VAS for health (r = 0.425, *P* < 0.001), and moderately with the VAS for QoL (r = 0.561, *P* < 0.001), WHOQOL-OLD (r = 0.656, *P* < 0.001) and CASP-12 (r = 0.663, *P* < 0.001). QoL is a multidimensional concept, so the low correlation with the EQ-5D-5L and its VAS could be explained by the fact that EQ-5D-5L is health-centred and does not explore any other dimensions, as suggested in the literature [76]. The correlation between the Swiss French version of the OPQOL and the WHOQOL-OLD was relatively similar to that of the original version (r = 0.698) assessed in a population of English origin (ONS Omnibus) by Bowling [22]. The correlation between the Swiss French version of the OPQOL and the CASP-12 was slightly lower than the one demonstrated by Bowling in her study using the CASP-19 (r = 0.732). However, the overall OPQOL score was statistically significant in correlation with validated questionnaires measuring QoL. This supports the convergent validity of the Swiss French OPQOL.

Cronbach’s alpha for the total scale was 0.875 for the test and 0.902 for the retest. That shows good internal consistency as the original English version (0.876 in the ONS Omnibus and 0.901 in the Follow-up) [22]. The internationally used OPQOL questionnaire also demonstrated very good internal consistency: 0.78 in Italy [13], 0.81 in Ghana [77], 0.834 in Sri Lanka [78], 0.90 in China [26] and 0.92 in Iran [24]. Considering the literature on the internal reliability of a questionnaire, the Swiss French version has a very acceptable reliability, neither too low nor too high [46, 62, 66].

The ICC_2.1_ of the OPQOL-35-SF total score indicates good test–retest reliability for research purposes, with values > 0.75 (total sample: 0.83, 95% CI 0.78–0.87; reduced sample: 0.83, 95% CI 0.77–0.87) [69]. Because the ICC_2.1_ was not > 0.9, it cannot be used individually [79]. The results obtained in the OPQOL-35-SF are slightly lower than those in the Chinese (ICC 0.87) and Persian (ICC 0.92) versions. The test–retest reliability of the original English version showed Spearman’s rho between 0.403 and 0.782. The test–retest reliability of subscales of the OPQOL-35-SF can be compared with the results of the Chinese and Persian versions. In the Swiss French version, two subscales showed an ICC_2.1_ between 0.75 and 0.9 and six subscales showed an ICC_2.1_ between 0.5 and 0.75; in the Chinese version, four subscales had an ICC between 0.75 and 0.9 and four subscales had an ICC between 0.5 and 0.75. However, the Persian version showed better results with four subscales having an ICC > 0.9, and four subscales having an ICC between 0.75 and 0.9. These differences could be due to the variation in time periods between completing the questionnaires (1–3 weeks for the Swiss French version, 4 weeks for the English version, and 2 weeks for the Chinese and Persian versions). The time between administration of the two questionnaires should be long enough to prevent participants from remembering what they had written, but short enough to prevent a change in the person’s situation [46]. It seems that with older people, a short duration is more appropriate [22]. The statistical methods used also differ between studies. ICC_2.1_ was used for the Swiss French version, Spearman’s rho for the English version, and ICC for the Chinese and Persian versions. It is possible that ICC_2.1_ might indicate a lower level of reliability compared with ICC [69]. For a positive rating for reliability, the weighted Kappa should be at least 0.70 [46]. Following the ratings of Landis & Koch [80], PABAK results between 0.80 and 1.00 indicate a “near-perfect agreement”; 0.60–0.79 “substantial agreement”, and 0.40–0.59 a “moderate agreement”. In OPQOL-35-SF, 17 items reached “near-perfect agreement”, and 18 items can be interpreted as “substantial agreement”. Six items had a PABAK < 0.70: Q6 “*I look forward to things*”, Q12 “*I’d like more people to enjoy life with*”, Q16 “*I do paid or unpaid work or activities that give me a role in life*”, Q19 *“The cost of the things compared to my pension/income restricts my life”*, Q21 “*I have responsibilities to others that restrict my social or leisure activities*”, and Q33 “*I cannot afford to do things I would enjoy*”. This may be explained by the participants’ reactions. Q6 was not easily understood; the participants did not know if the item referred to the present moment or in general. Participants took a long time to answer Q12 because the coding is reversed. Q19 and Q21 often needed clarifications. Q33 follows a similar item, but is expressed in positive terms. Participants took more time for the last questions, because of loss of concentration. The original version of OPQOL contains eight items with a voluntary reversed scoring, to avoid the participants automatically selecting the same reply [81]. The relevance of reverse coding is discussed [82, 83]. In the Czech translation of the questionnaire, the rating was reversed in order to respond to local and socio-cultural practice, i.e. the "best rating" is 1 and the "worst rating" is 5 [25].

For the current study, SC and CM were trained to conduct “one-to-one” and “in-group” interviews. This enabled informal recording of participants' experiences when completing the questionnaires. Some participants would have liked "memory" to be the subject of an item, as memory loss is a concern for older adults. Many participants would have liked religion and culture to have been differentiated in the items. Most participants would have preferred to be able to give "yes" or "no" answers. The choice of 5 answers offered by the Likert scale was not easy to integrate; perhaps a 3-level scale should be considered for the elderly population. In addition, some participants would have liked to complete their answers using qualitative information.

This self-administered assessment of the QoL of older people could be completed under the supervision of a physiotherapist, during a session held either in the practice or at the patient’s home. Although the ICC test–retest reliability of the Swiss French version of the OPQOL-35 is not > 0.90, this tool might inspire physiotherapists to learn about the QoL of their older patients, and provide information that would be valuable in improving bio-psycho-social care.

### Strengths and limitations

The OPQOL-35-SF questionnaire had good acceptance in the study sample; only two participants declined to participate in the second session (retest). Another strength of this study was the completeness of data collection. Rigorous supervision during completion of the questionnaires ensured that missing data was very limited.

A possible limitation in the data collection was the transfer from paper into digital format, which may introduce human error despite all precautions being applied.

Another limitation is the homogeneity of our population. Indeed, most of the participants were active, in fit and engaged in social activities. This might limit the generalizability of the results; no conclusion can be drawn on the validity and reliability of the OPQOL-35-SF for a population in poorer health or poorer condition. Since not all the participants completed the questionnaire in the same settings (i.e., they were either in a group session or individual session with one supervisor), the impact of this on the answers is unknown. It is possible that participants in group may have been embarrassed or afraid to give negative answers.

### Further research

It would be of interest to extend this study by recruiting 200 additional individuals to perform a CFA in order to test the new redistribution of the items to factors in the French translation. The authors of this study translated and assessed the psychometric properties of the Swiss French version of the OPQOL-35 for use in Switzerland. However, french speakers represent only 25% of the Swiss population; Switzerland has four national languages and German is spoken by more than 64% of its population. To our knowledge, the OPQOL-35 has not been translated or validated in German; this could therefore be a subject for further research.

## Conclusion

The Swiss French version of the OPQOL-35 questionnaire (OPQOL-35-SF) shows good reliability and construct validity. These results permit its use to evaluate QoL in older people in clinical practice or research. However, we recommend applying the questionnaire under the supervision of a health professional in order to reduce the number of missing items. The questionnaire is freely available under: https://www.hevs.ch/en/projects/validation-of-questionnaires-201777/, in the “Documents” section. Future research should explore the use of a supplementary sample to perform a CFA and gain a better distribution of the items in the different factors.

## Supplementary Information

**Additional file 1**. Older People Quality of Life Questionnaire 35 Swiss French Version (OPQOL-35-SF) used in the current study. Translated OPQOL-35 in Swiss French

**Additional file 2**. Score conversions. Table displaying the equation of the conversion of the score of the questionnaires to meet the range of scores of the OPQOL.

**Additional file 3**. Principal Component Analysis (test). Table displaying the detailed results of the PCA for the test

**Additional file 4**. Principal Component Analysis (retest). Table displaying the detailed results of the PCA for the retest

**Additional file 5**. Final Version of Older People Quality of Life Questionnaire 35 Swiss French translation (OPQOL-35-SF). OPQOL-35-SF resulting from the study

**Additional file 6**. Cohen’s kappa and prevalence-adjusted bias-adjusted kappa (PABAK). Table displaying the detailed results of the Cohen’s kappa and PABAK separated for the total sample and the reduced sample

## Data Availability

The datasets used and/or analysed during the current study are available from the corresponding author on reasonable request.

## Abbreviations

CASP: Control, Autonomy, Self-realization, and Pleasure
CASP-12: Control, Autonomy, Self-realization, and Pleasure in 12 questions
CFA: Confirmatory factor analysis
EFA: Explanatory factor analysis
EQ: EuroQol
EQ-5D-5L: EuroQol-5 dimensions-5- levels
EUROHIS-QOL: European Health Interview Survey-Quality of Life
ICC: Intra-class correlation coefficient
ICC_2.1_: Intra-class correlation coefficient, two-way random effects, absolute agreement, single rater
KMO: Kaiser–Meyer–Olkin measure of sampling adequacy
OPQOL: Older People’s Quality of Life questionnaire
OPQOL-35: Older People’s Quality of Life questionnaire, 35 questions
OPQOL-35-SF: Swiss French version of the OPQOL-35 questionnaire
PABAK: Prevalence-adjusted bias-adjusted kappa
PCA: Principal component analysis
QoL: Quality of life
REDCap: Research Electronic Data Capture
TV: Transformed values
UN: United Nations
VAS: Visual Analogue Scale
WHO: World Health Organisation
WHOQOL-AGE: World Health Organisation Quality of Life in the ageing population questionnaire
WHOQOL-OLD: World Health Organisation Quality of Life in older people questionnaire
