# Supplementary material for: Evaluation of the psychometric properties of the Swiss French version of the Older People’s Quality of Life questionnaire (OPQOL-35-SF)
Source: Health Qual Life Outcomes. 2022 Mar 9;20:43. doi: 10.1186/s12955-022-01950-w (PMC8905913; doi:10.1186/s12955-022-01950-w)
Supplement: Supplementary file 1 — Additional file 1. Older People Quality of Life Questionnaire 35 Swiss French Version (OPQOL-35-SF) used in the current study. Translated OPQOL-35 in Swiss French [file 12955_2022_1950_MOESM1_ESM.pdf]

Additional material 1 : Older people's quality of life questionnaire Swiss French version used in the current study

OPQOL-35-SF

QUESTIONNAIRE SUR LA QUALITÉ DE VIE DES PERSONNES ÂGÉES (OPQOL-35)

Nous aimerions vous interroger sur votre qualité de vie :

Aujourd'hui, en ce qui concerne votre qualité de vie :

	Très mauvaise qualité de vie	Excellente qualité de vie
Veuillez indiquer sur l'échelle ci-dessous, comment vous évaluez en ce moment votre qualité de vie	☐☐	

Veuillez choisir une seule proposition de réponse par ligne. Il n'y a ni réponses justes ni réponses fausses. Veuillez choisir la réponse qui correspond le mieux à ce que vous ressentez.

1. Considérant les aspects positifs et négatifs de votre vie, comment décririez-vous votre qualité de vie dans son ensemble ?

Dans l'ensemble, votre qualité de vie est:

- O Très bonne
- O Bonne
- O Acceptable
- O Mauvaise
- O Très mauvaise

2. Veuillez indiquer le degré d'accord qui correspond au mieux à ce que vous ressentez. Choisissez une seule proposition de réponse par ligne

La vie en général

	Tout à fait d'accord	D'accord	Ni d'accord, ni pas d'accord	Pas d'accord	Pas du tout d'accord
(1) Dans l'ensemble, je suis satisfait(e) de ma vie	O	O	O	O	O
(2) Je suis heureux-se la plupart du temps	O	O	O	O	O
(3) Je me réjouis de ce que la vie peut m'offrir	O	O	O	O	O
(4) La vie me déprime	O	O	O	O	O

La santé

	Tout à fait d'accord	D'accord	Ni d'accord, ni pas d'accord	Pas d'accord	Pas du tout d'accord
(5) Physiquement, j'ai beaucoup d'énergie	O	O	O	O	O
(6) La douleur affecte mon bien être	O	O	O	O	O
(7) Mon état de santé m'empêche de m'occuper de moi ou de mon foyer	O	O	O	O	O
(8) Je suis suffisamment en bonne santé pour sortir de chez moi	O	O	O	O	O

Relations sociales et participation à la vie sociale

	Tout à fait d'accord	D'accord	Ni d'accord, ni pas d'accord	Pas d'accord	Pas du tout d'accord
(9) Ma famille, mes amis ou mes voisins m'aideraient en cas de besoin	O	O	O	O	O
(10) J'aimerais plus de compagnie ou de contacts avec d'autres personnes	O	O	O	O	O
(11) J'ai quelqu'un qui me donne de l'amour et de l'affection	O	O	O	O	O
(12) J'aimerais être plus entouré-e pour apprécier la vie	O	O	O	O	O
(13) Mes enfants sont proches de moi, c'est important (Si pas d'enfants, proposez neveux ou nièces ou autres personnes plus jeunes et proches)	O	O	O	O	O
(14) J'ai des activités sociales et des loisirs qui me plaisent	O	O	O	O	O
(15) J'essaie de continuer à être actif-ve	O	O	O	O	O
(16) Je poursuis des activités bénévoles ou rémunérées qui donnent un sens à ma vie	O	O	O	O	O

Indépendance, maîtrise de sa vie et liberté

	Tout à fait d'accord	D'accord	Ni d'accord, ni pas d'accord	Pas d'accord	Pas du tout d'accord
(17) Je suis suffisamment en bonne santé pour être indépendant-e	O	O	O	O	O
(18) J'éprouve du plaisir à ce que je fais	O	O	O	O	O
(19) Le coût de la vie par rapport à mes revenus réduit mon niveau de vie	O	O	O	O	O
(20) Je maîtrise bien les éléments importants de ma vie	O	O	O	O	O
(21) J'ai des responsabilités envers d'autres qui restreignent mes activités sociales ou mes loisirs	O	O	O	O	O

Domicile et voisinage

	Tout à fait d'accord	D'accord	Ni d'accord, ni pas d'accord	Pas d'accord	Pas du tout d'accord
(22) Je me sens en sécurité où je vis	O	O	O	O	O
(23) Les infrastructures, les commerces et les services locaux sont généralement bons.	O	O	O	O	O
(24) J'ai du plaisir à être chez moi	O	O	O	O	O
(25) Je trouve mon voisinage sympathique	O	O	O	O	O

Le bien-être psychologique et émotionnel

	Tout à fait d'accord	D'accord	Ni d'accord, ni pas d'accord	Pas d'accord	Pas du tout d'accord
(26) Je prends la vie comme elle vient et je fais au mieux	O	O	O	O	O
(27) Je me sens chanceux-se par rapport à la plupart des gens	O	O	O	O	O
(28) En général, je vois plutôt le bon côté des choses	O	O	O	O	O
(29) Si mon état de santé devait limiter mes activités sociales et mes loisirs, je compenserais en trouvant d'autres activités	O	O	O	O	O

Situation financière

	Tout à fait d'accord	D'accord	Ni d'accord, ni pas d'accord	Pas d'accord	Pas du tout d'accord
(30) J'ai assez d'argent pour payer les factures du ménage	O	O	O	O	O
(31) J'ai assez d'argent pour payer l'entretien de mon logement ou de mon ménage	O	O	O	O	O
(32) J'ai l'argent nécessaire pour acheter ce dont j'ai envie	O	O	O	O	O
(33) Je n'ai pas l'argent nécessaire pour faire ce qui me plairait	O	O	O	O	O

Culture et religion

	Tout à fait d'accord	D'accord	Ni d'accord, ni pas d'accord	Pas d'accord	Pas du tout d'accord
(34) La religion, la foi ou la philosophie sont importantes pour ma qualité de vie	O	O	O	O	O
(35) Assister à des évènements religieux ou culturels est important pour ma qualité de vie	O	O	O	O	O
