# Supplementary material for: Evaluation of the psychometric properties of the Swiss French version of the Older People’s Quality of Life questionnaire (OPQOL-35-SF)
Source: Health Qual Life Outcomes. 2022 Mar 9;20:43. doi: 10.1186/s12955-022-01950-w (PMC8905913; doi:10.1186/s12955-022-01950-w)
Supplement: Supplementary file 2 — Additional file 2. Score conversions. Table displaying the equation of the conversion of the score of the questionnaires to meet the range of scores of the OPQOL. [file 12955_2022_1950_MOESM2_ESM.pdf]

## Additional material 2: Score conversions

| | | OPQOL-35 | VAS QoL/VAS Health | WHOQOL-OLD | CASP-12 | EQ-5D-5L |
|---|---|---|---|---|---|---|
| Original Questionnaires | ***Number of items*** | 35 | - | 24 | 12 | 5 |
| | ***Scoring*** | 1-5 | - | 1-5 | 0-3 | Algorithme |
| | ***Minimum total score*** | 35 | 0 | 24 | 0 | -0.53 |
| | ***Maximum total score*** | 175 | 100 | 120 | 36 | 1 |
| **Range** | | 140 | 100 | 96 | 36 | 1.53 |
| | ***Coefficient*** | - | 1.4 | 1.458333 | 3.88889 | 91.503268 |
| | ***Conversion*** | | +35 | | +35 | +0.53 / +35 |
| Verification | ***Minimum total score*** | 35 | 35 | 35 | 35 | 35 |
| | ***Maximum total score*** | 175 | 175 | 175 | 175 | 175 |

*OPQOL-35: Older People's Quality of Live Questionnaire; VAS QoL: Visual Analogue Scale for Quality of Live; VAS health: Visual Analogue Scale for health; WHOQOL-OLD: World Health Organization Quality of Life in older people questionnaire; CASP-12: Control, Autonomy, Self-realization, Pleasure in 12 questions; EQ-5D-5L: EuroQol-5-dimensions-5- levels.*
