# Supplementary material for: Evaluation of the psychometric properties of the Swiss French version of the Older People’s Quality of Life questionnaire (OPQOL-35-SF)
Source: Health Qual Life Outcomes. 2022 Mar 9;20:43. doi: 10.1186/s12955-022-01950-w (PMC8905913; doi:10.1186/s12955-022-01950-w)
Supplement: Supplementary file 3 — Additional file 3. Principal Component Analysis (test). Table displaying the detailed results of the PCA for the test [file 12955_2022_1950_MOESM3_ESM.pdf]

Additional material 3: Principal Component Analysis (test)

Principal Component Analysis test (n= 264)

Question's number and items		***RC1***	***RC2***	***RC3***	***RC4***	***RC5***	***RC6***	***RC7***	***RC8***
1	I enjoy my life overall	0.70							
2	I am happy much of the time	0.77							
3	I look forward to things	0.72							
4	Life gets me down	0.63							
5	I have a lot of physical energy			0.61					
6	Pain affects my well-being			0.39			-0.44		
7	My health restricts me looking after myself or my home			0.55				0.30	
8	I am healthy enough to get out and about							0.52	0.38
9	My family, friends or neighbours would help me if needed								0.57
10	I would like more companionship or contact with other people				0.78				
11	I have someone who gives me love and affection	0.31				0.32			0.38
12	I'd like more people to enjoy life with				0.75				
13	I have my children around which is important								0.70
14	I have social or leisure activities/hobbies that I enjoy doing			0.61			0.34		
15	I try to stay involved with things			0.73					
16	I do paid or unpaid work or activities that give me a role in life			0.70					
17	I am healthy enough to have my independence			0.62				0.35	
18	I can please myself what I do	0.51		0.43			0.35		
19	The cost of things compared to my pension/income restricts my life		0.72						
20	I have a lot of control over the important things in my life	0.50		0.38					
21	I have responsibilities to others that restrict my social or leisure activities				0.61				
22	I feel safe where I live							0.67	
23	The local shops, services and facilities are good overall						0.60		
24	I get pleasure from my home	0.32					0.31	0.64	
25	I find my neighbourhood friendly						0.36		
26	I take life as it comes and make the best of things	0.59					0.32		
27	I feel lucky compared to most people	0.36					0.47		
28	I tend to look on the bright side	0.61					0.44		
29	If my health limits social/leisure activities, then I will compensate and find something else I can do			0.52					
30	I have enough money to pay for household bills		0.73						
31	I have enough money to pay for household repairs or help needed in the house		0.75						
32	I can afford to buy what I want to		0.75						
33	I cannot afford to do things I would enjoy		0.69						
34	Religion, belief or philosophy is important to my quality of life					0.89			
35	Cultural/religious events/festivals are important to my quality of life					0.89			
Explained Proportion		0.21	0.15	0.18	0.10	0.09	0.10	0.09	0.08
