# Supplementary material for: Evaluation of the psychometric properties of the Swiss French version of the Older People’s Quality of Life questionnaire (OPQOL-35-SF)
Source: Health Qual Life Outcomes. 2022 Mar 9;20:43. doi: 10.1186/s12955-022-01950-w (PMC8905913; doi:10.1186/s12955-022-01950-w)
Supplement: Supplementary file 4 — Additional file 4. Principal Component Analysis (retest). Table displaying the detailed results of the PCA for the retest [file 12955_2022_1950_MOESM4_ESM.pdf]

Additional material 4: Principal Component Analysis (retest)

Principal Component Analysis retest (n= 238)

Question's number and items		rRC1	rRC2	rRC3	rRC4	rRC5	rRC6	rRC7	rRC8
1	I enjoy my life overall								0.72
2	I am happy much of the time								0.76
3	I look forward to things					0.31			0.69
4	Life gets me down								0.61
5	I have a lot of physical energy					0.58			
6	Pain affects my well-being				0.56	0.43			
7	My health restricts me looking after myself or my home					0.67			
8	I am healthy enough to get out and about					0.56			
9	My family, friends or neighbours would help me if needed	0.42					0.48		
10	I would like more companionship or contact with other people							0.83	
11	I have someone who gives me love and affection						0.55		0.39
12	I'd like more people to enjoy life with							0.75	
13	I have my children around which is important						0.74		
14	I have social or leisure activities/hobbies that I enjoy doing	0.59				0.40			
15	I try to stay involved with things	0.59				0.41			
16	I do paid or unpaid work or activities that give me a role in life					0.45			
17	I am healthy enough to have my independence	0.32				0.73			
18	I can please myself what I do	0.55				0.47			
19	The cost of things compared to my pension/income restricts my life		0.78						
20	I have a lot of control over the important things in my life	0.44				0.48			
21	I have responsibilities to others that restrict my social or leisure activities				-0.47			0.36	0.30
22	I feel safe where I live	0.38			-0.37	0.35	0.35		
23	The local shops, services and facilities are good overall	0.63							
24	I get pleasure from my home	0.39					0.43		
25	I find my neighbourhood friendly	0.36					0.63		
26	I take life as it comes and make the best of things	0.51							0.32
27	I feel lucky compared to most people	0.52							
28	I tend to look on the bright side	0.59							0.37
29	If my health limits social/leisure activities, then I will compensate and find something else I can do	0.55			0.34				
30	I have enough money to pay for household bills		0.72			0.32			
31	I have enough money to pay for household repairs or help needed in the house		0.71						
32	I cannot afford to do things I would enjoy		0.80						
33	I cannot afford to do things I would enjoy		0.74						
34	Religion, belief or philosophy is important to my quality of life			0.89					
35	Cultural/religious events/festivals are important to my quality of life			0.89					
Explained Proportion		0.19	0.16	0.10	0.06	0.16	0.11	0.09	0.19
