# Supplementary material for: Evaluation of the psychometric properties of the Swiss French version of the Older People’s Quality of Life questionnaire (OPQOL-35-SF)
Source: Health Qual Life Outcomes. 2022 Mar 9;20:43. doi: 10.1186/s12955-022-01950-w (PMC8905913; doi:10.1186/s12955-022-01950-w)
Supplement: Supplementary file 5 — Additional file 5. Final Version of Older People Quality of Life Questionnaire 35 Swiss French translation (OPQOL-35-SF). OPQOL-35-SF resulting from the study [file 12955_2022_1950_MOESM5_ESM.pdf]

Additional material 5 : Final version Older People’s Quality of Life Questionnaire (OPQOL-35-SF)

Older People’s Quality of Life Questionnaire (OPQOL-35-SF)

QUESTIONNAIRE SUR LA QUALITÉ DE VIE DES PERSONNES ÂGÉES (OPQOL-35-SF)

Nous aimerions vous interroger sur votre qualité de vie :

Aujourd'hui, en ce qui concerne votre qualité de vie :

Veuillez indiquer sur l'échelle ci-dessous, comment vous évaluez en ce moment votre qualité de vie

Très mauvaise qualité de vie	Excellente qualité de vie

Veuillez choisir une seule proposition de réponse par ligne. Il n'y a ni réponses justes ni réponses fausses. Veuillez choisir la réponse qui correspond le mieux à ce que vous ressentez.

1. Considérant les aspects positifs et négatifs de votre vie, comment décririez-vous votre qualité de vie dans son ensemble ?

Dans l'ensemble, votre qualité de vie est:

- O Très bonne
- O Bonne
- O Acceptable
- O Mauvaise
- O Très mauvaise

2. Veuillez indiquer le degré d'accord qui correspond au mieux à ce que vous ressentez. Choisissez une seule proposition de réponse par ligne

Bien-être psychologique et émotionnel

	Tout à fait d'accord	D'accord	Ni d'accord, ni pas d'accord	Pas d'accord	Pas du tout d'accord
(1) Dans l'ensemble, je suis satisfait(e) de ma vie	O	O	O	O	O
(2) Je suis heureux-se la plupart du temps	O	O	O	O	O
(3) Je me réjouis de ce que la vie peut m'offrir	O	O	O	O	O
(4) La vie me déprime	O	O	O	O	O
(5) Je prends la vie comme elle vient et je fais au mieux	O	O	O	O	O
(6) Je me sens chanceux-se par rapport à la plupart des gens	O	O	O	O	O
(7) En général, je vois plutôt le bon côté des choses	O	O	O	O	O

Situation financière

	Tout à fait d'accord	D'accord	Ni d'accord, ni pas d'accord	Pas d'accord	Pas du tout d'accord
(8) Le coût de la vie par rapport à mes revenus réduit mon niveau de vie	O	O	O	O	O
(9) J'ai assez d'argent pour payer les factures du ménage	O	O	O	O	O
(10) J'ai assez d'argent pour payer l'entretien de mon logement ou de mon ménage	O	O	O	O	O
(11) J'ai l'argent nécessaire pour acheter ce dont j'ai envie	O	O	O	O	O
(12) Je n'ai pas l'argent nécessaire pour faire ce qui me plairait	O	O	O	O	O

Condition physique

	Tout à fait d'accord	D'accord	Ni d'accord, ni pas d'accord	Pas d'accord	Pas du tout d'accord
(13) Physiquement, j'ai beaucoup d'énergie	O	O	O	O	O
(14) La douleur affecte mon bien être	O	O	O	O	O
(15) Mon état de santé m'empêche de m'occuper de moi ou de mon foyer	O	O	O	O	O
(16) J'ai des activités sociales et des loisirs qui me plaisent	O	O	O	O	O
(17) J'essaie de continuer à être actif-ve	O	O	O	O	O
(18) Je poursuis des activités bénévoles ou rémunérées qui donnent un sens à ma vie	O	O	O	O	O
(19) Je suis suffisamment en bonne santé pour être indépendant-e	O	O	O	O	O
(20) J'éprouve du plaisir à ce que je fais	O	O	O	O	O
(21) Je maîtrise bien les éléments importants de ma vie	O	O	O	O	O

Relations sociales

	Tout à fait d'accord	D'accord	Ni d'accord, ni pas d'accord	Pas d'accord	Pas du tout d'accord
(22) J'aimerais plus de compagnie ou de contacts avec d'autres personnes	O	O	O	O	O
(23) J'aimerais être plus entouré-e pour apprécier la vie	O	O	O	O	O
(24) J'ai des responsabilités envers d'autres qui restreignent mes activités sociales ou mes loisirs	O	O	O	O	O

Culture et religion

	Tout à fait d'accord	D'accord	Ni d'accord, ni pas d'accord	Pas d'accord	Pas du tout d'accord
(25) La religion, la foi ou la philosophie sont importantes pour ma qualité de vie	O	O	O	O	O
(26) Assister à des évènements religieux ou culturels est important pour ma qualité de vie	O	O	O	O	O

Domicile et voisinage

	Tout à fait d'accord	D'accord	Ni d'accord, ni pas d'accord	Pas d'accord	Pas du tout d'accord
(27) Les infrastructures, les commerces et les services locaux sont généralement bons.	O	O	O	O	O
(28) J'ai du plaisir à être chez moi	O	O	O	O	O
(29) Je trouve mon voisinage sympathique	O	O	O	O	O

Contexte familial

	Tout à fait d'accord	D'accord	Ni d'accord, ni pas d'accord	Pas d'accord	Pas du tout d'accord
(31) Ma famille, mes amis ou mes voisins m'aideraient en cas de besoin	O	O	O	O	O
(31) J'ai quelqu'un qui me donne de l'amour et de l'affection	O	O	O	O	O
(32) Mes enfants sont proches de moi, c'est important (Si pas d'enfants, proposez neveux ou nièces ou autres personnes plus jeunes et proches)	O	O	O	O	O

Autres

	Tout à fait d'accord	D'accord	Ni d'accord, ni pas d'accord	Pas d'accord	Pas du tout d'accord
(33) Je suis suffisamment en bonne santé pour sortir de chez moi	O	O	O	O	O
(34) Je me sens en sécurité où je vis	O	O	O	O	O
(35) Si mon état de santé devait limiter mes activités sociales et mes loisirs, je compenserais en trouvant d'autres activités	O	O	O	O	O
