# Supplementary material for: Evaluation of the psychometric properties of the Swiss French version of the Older People’s Quality of Life questionnaire (OPQOL-35-SF)
Source: Health Qual Life Outcomes. 2022 Mar 9;20:43. doi: 10.1186/s12955-022-01950-w (PMC8905913; doi:10.1186/s12955-022-01950-w)
Supplement: Supplementary file 6 — Additional file 6. Cohen’s kappa and prevalence-adjusted bias-adjusted kappa (PABAK). Table displaying the detailed results of the Cohen’s kappa and PABAK separated for the total sample and the reduced sample [file 12955_2022_1950_MOESM6_ESM.pdf]

Additional material 6: Cohen's kappa and prevalence-adjusted bias-adjusted kappa (PABAK)

Item	Full sample (n= 262)			Participant without extra events (n=238)		
	AG. (%)	*Kappa (weighted) (95% CI)*	*PABAK (95% CI)*	*AG. (%)*	*Kappa (weighted) (95% CI)*	*PABAK (95% CI)*
1	91.8	0.49 (0.39-0.58)	0.84 (0.79-0.88)	92.1	0.5 (0.39-0.59)	0.84 (0.81-0.88)
2	90.6	0.44 (0.34-0.54)	0.81 (0.76-0.86)	90.4	0.43 (0.33-0.53)	0.81 (0.77-0.86)
3	87.5	0.44 (0.34-0.53)	0.75 (0.7-0.79)	87.5	0.42 (0.33-0.52)	0.75 (0.7-0.8)
4	87.4	0.37 (0.28-0.46)	0.75 (0.69-0.8)	87.7	0.36 (0.25-0.46)	0.75 (0.69-0.81)
5	88.5	0.47 (0.39-0.55)	0.77 (0.7-0.81)	88.4	0.46 (0.37-0.55)	0.77 (0.69-0.81)
6	83.2	0.48 (0.41-0.55)	0.66 (0.62-0.71)	83.2	0.48 (0.4-0.56)	0.66 (0.61-0.71)
7	86.5	0.41 (0.31-0.5)	0.73 (0.69-0.78)	87.4	0.43 (0.33-0.52)	0.75 (0.69-0.79)
8	88.5	0.25 (0.14-0.35)	0.77 (0.71-0.83)	87.8	0.22 (0.12-0.34)	0.76 (0.69-0.81)
9	90.7	0.44 (0.33-0.54)	0.81 (0.75-0.85)	90.4	0.43 (0.33-0.53)	0.81 (0.76-0.86)
10	84.9	0.51 (0.41-0.58)	0.7 (0.65-0.75)	84.9	0.51 (0.41-0.59)	0.7 (0.64-0.75)
11	89.3	0.44 (0.35-0.53)	0.79 (0.74-0.83)	89.4	0.44 (0.34-0.54)	0.79 (0.74-0.83)
12	83.6	0.49 (0.4-0.57)	0.67 (0.62-0.72)	83.5	0.49 (0.4-0.58)	0.67 (0.62-0.72)
13	92.6	0.58 (0.47-0.68)	0.85 (0.81-0.89)	93.3	0.62 (0.51-0.71)	0.87 (0.82-0.9)
14	91.6	0.5 (0.41-0.6)	0.83 (0.78-0.87)	91.7	0.48 (0.39-0.58)	0.83 (0.79-0.9)
15	92.6	0.49 (0.4-0.58)	0.85 (0.81-0.89)	93	0.52 (0.42-0.62)	0.86 (0.82-0.9)
16	83.5	0.58 (0.5-0.65)	0.67 (0.61-0.72)	83.1	0.56 (0.47-0.63)	0.66 (0.61-0.72)
17	92.2	0.49 (0.38-0.6)	0.84 (0.79-0.87)	92	0.49 (0.38-0.6)	0.84 (0.79-0.88)
18	92.5	0.47 (0.36-0.57)	0.85 (0.76-0.89)	92.5	0.46 (0.36-0.57)	0.85 (0.79-0.89)
19	81.6	0.48 (0.39-0.57)	0.63 (0.56-0.69)	81.6	0.48 (0.39-0.57)	0.63 (0.57-0.69)
20	88.5	0.39 (0.28-0.48)	0.77 (0.71-0.81)	89.1	0.41 (0.32-0.52)	0.78 (0.71-0.82)
21	83	0.4 (0.32-0.49)	0.66 (0.61-0.71)	82.7	0.4 (0.31-0.5)	0.65 (0.6-0.71)
22	92.7	0.47 (0.35-0.6)	0.85 (0.79-0.9)	92.6	0.46 (0.32-0.6)	0.85 (0.77-0.9)
23	90.6	0.58 (0.5-0.66)	0.81 (0.77-0.85)	90.7	0.58 (0.49-0.67)	0.81 (0.77-0.85)
24	90.1	0.47 (0.36-0.6)	0.8 (0.61-0.85)	89.9	0.45 (0.31-0.56)	0.8 (0.59-0.87)
25	91.9	0.54 (0.45-0.62)	0.84 (0.78-0.88)	92.1	0.56 (0.48-0.65)	0.84 (0.8-0.89)
26	88.2	0.38 (0.28-0.48)	0.76 (0.68-0.81)	88.4	0.37 (0.25-0.48)	0.77 (0.66-0.81)
27	90.9	0.44 (0.34-0.53)	0.82 (0.75-0.87)	91.3	0.45 (0.35-0.55)	0.83 (0.77-0.88)
28	90.6	0.47 (0.37-0.56)	0.81 (0.76-0.85)	90.4	0.45 (0.34-0.55)	0.81 (0.76-0.85)
29	87.3	0.44 (0.36-0.52)	0.75 (0.7-0.78)	87.2	0.43 (0.34-0.51)	0.74 (0.7-0.79)
30	92.3	0.53 (0.43-0.62)	0.85 (0.8 à 0.89)	91.6	0.5 (0.39-0.59)	0.83 (0.78-0.87)
31	92.2	0.52 (0.43-0.61)	0.84 (0.79-0.89)	91.6	0.49 (0.4-0.59)	0.83 (0.77-0.87)
32	88.4	0.56 (0.47-0.63)	0.77 (0.73-0.81)	88.2	0.56 (0.47-0.63)	0.76 (0.72-0.8)
33	83.1	0.46 (0.38-0.54)	0.66 (0.6-0.71)	83.4	0.47 (0.38-0.56)	0.67 (0.61-0.72)
34	91.9	0.7 (0.63-0.75)	0.84 (0.81-0.87)	91.4	0.67 (0.6-0.73)	0.83 (0.79-0.86)
35	88.5	0.58 (0.51-0.65)	0.77 (0.73-0.8)	88.2	0.56 (0.49-0.63)	0.76 (0.72-0.8)

AG. (%): Agreement in percent; CI: Confidence Interval; PABAK: Prevalence-Adjusted Bias-Adjusted Kappa
